# Enrofloxacin in Poultry and Human Health

**DOI:** 10.3201/eid1205.051477

**Published:** 2006-05

**Authors:** Louis Anthony Cox

**Affiliations:** * Cox Associates, Denver, Colorado, USA;; † University of Colorado Health Sciences Center, Denver, Colorado, USA

**Keywords:** fluoroquinolones, drug resistance, risk assessment, letter

**To the Editor:** Following logic similar to that recently used by the US Food and Drug Administration to withdraw approval for enrofloxacin, a recent letter estimated that fluoroquinolone use in poultry could compromise responses to antimicrobial drugs in >24,000 persons per year in the United States (*1*). However, >99.9% of this estimated risk appears to result from incorrect assumptions. Potentially important corrections include the following: 1) not attributing resistance from foreign travel and human ciprofloxacin use to domestic use of enrofloxacin in poultry (this could reduce the estimated risk by ≈1/3) (*2*); 2) updating the estimated fraction of human foodborne *Campylobacter* infections caused by poultry to reflect declines in microbial loads on chicken carcasses since 1992 reduces the estimated risk by a factor of perhaps 1/10 (*3*) (the cited 90% estimate by Hurd et al. [1] was intended for use as part of a conservative upper-bounding analysis, not as a realistic point estimate); 3) replacing an assumption that 10% of infected persons would benefit from antimicrobial drug therapy with a more data-based value of 0.6% (*4*) would reduce the estimated risk by a factor of 0.6/10 = 0.06; 4) replacing an assumption that fluoroquinolones are prescribed for *all* affected patients receiving antimicrobial drug treatment (rather than, for example, erythromycin) by a more realistic value of fluoroquinolones being prescribed for perhaps ≈50% of patients (*2*) reduces the estimated risk by a factor of ≈50%; 5) replacing an assumption that *all* such cases lead to compromised responses with a more data-driven estimate that perhaps ≈17% of patients have compromised responses would reduce the estimated risk by a factor of 1/6 (*5*); and 6) recognizing that reducing enrofloxacin use may not decrease fluoroquinolone resistance in all *Campylobacter* spp. from food animals (effect not quantified) (*6*). Together, such changes reduce the estimated risk by a factor of at least (1/3) × (1/10) × (0.6/10) × (1/2) × (1/6) = 0.00017, or by >99.9%.

More notably, the calculation in (*1*) also wrongly assumes that the fraction of patients with fluoroquinolone-resistant infections times the fraction of infections caused by poultry gives the fraction of patients with compromised response caused by fluoroquinolone use in poultry. As a simple counterexample, suppose that 80% of all infections were caused by poultry, with the rest caused by something else (e.g., water), and that all and only the 20% of infections caused by the latter source are resistant. Then the procedure in (*1*) would estimate (80% of infections caused by poultry) × (20% of infections resistant) = 16% as the fraction of resistant infections caused by poultry, even though the correct answer is zero. Thus, the basic logic of the calculation is flawed.
