# Supplementary material for: Enrofloxacin in Poultry and Human Health
Source: Emerg Infect Dis. 2006 May;12(5):872–3. doi: 10.3201/eid1205.051477 (PMC3374429; doi:10.3201/eid1205.051477)
Supplement: Related texts

# Reply: Enrofloxacin in Poultry and Human Health

**Authors:** Peter Collignon

**Affiliations:** * The Canberra Hospital, Woden, Australian Capital Territory, Australia

**In response:** Cox's letter (*1*) contains a number of false assumptions, errors, misleading assertions, and misquotations. Cox asserts that annually 1 person or fewer in the United States will experience an adverse effect because of fluoroquinolone use in poultry. He reduces 10-fold my referenced risk for persons acquiring *Campylobacter* infections from poultry (*2*). His unrealistically low estimate is not given in his referenced citation. His estimated risk is also much lower than in the reference 2, which Cox himself quotes, "Poultry is the most common cause of sporadic cases of campylobacteriosis in the United States" (Economic Research Service of the US Department of Agriculture) (*3*). Cox knows that his assertion (*4*) that poultry make little or no contribution to human *Campylobacter* infections has been extensively examined and found to be wrong. Indeed, an entire section in a recent US Food and Drug Administration (FDA) determination was written about the unreliability of Cox's testimony and these assertions, a finding made by both the FDA commissioner and an administrative law judge (*5**,**6*).

Cox also misquotes Busby et al. (*3*) when he asserts that only 0.6% of persons with *Campylobacter* infections benefit from antimicrobial drugs. The Busby article states that 0.6% of persons with *Campylobacter* infections need "hospitalization," not how many would benefit from antimicrobial drug therapy. Cox has thus made a misleading attribution (something he has previously been found to do [5]).

Busby et al. (*3*) estimated that in 1993, ≈1,500,000 persons in the United States acquired *Campylobacter* infections from food sources. Even if the proportion who can benefit from receiving antimicrobial drugs is as low as 2%, this translates to 30,000 persons. If 20% of these infections were caused by fluoroquinolone-resistant *Campylobacter* spp., then 6,000 persons would potentially have their therapy and outcome compromised, rather than the 1 person that Cox would have us believe. More realistic is the figure of 24,000 persons estimated previously to be at risk of having an adverse outcome (or ≈285 persons for every 1 million chickens treated with fluoroquinolones) (*1*). Cox's assumptions and calculations thus seem flawed and unrealistic.

## References

[1] Cox LA. Enrofloxacin in poultry and human health [letter]. Emerg Infect Dis. 2006;12:872–3.10.3201/eid1205.05147716710963PMC3374429

[2] Collignon P. Fluoroquinolone use in food animals [letter]. Emerg Infect Dis. 2005;11:1789–90.1642200410.3201/eid1111.040630PMC3367372

[3] Busby JC, Roberts T, Jordan Lin C-T, MacDonald JC. Bacterial food-borne disease: medical costs and productivity losses. [cited 2006 Mar 8]. Agricultural Economics Report No. (AER741). Washington: United States Department of Agriculture; 1996. Available from http://www.ers.usda.gov/publications/aer741/

[4] Phillips I, Casewell M, Cox T, DeGroot B, Friis C, Jones RN, Does the use of antibiotics in food animals pose a risk to human health? A critical review of published data. J Antimicrob Chemother. 2004;53:28–52.10.1093/jac/dkg48314657094

[5] US Food and Drug Administration. Final decision of the commissioner. Proposal to withdraw the approval of the new animal drug application for enrofloxacin for poultry [cited 2006 Mar 8]. Docket no. 2000N-1571. p. 16–7; 108–19. Available from http://www.fda.gov/oc/antimicrobial/baytril.pdf

[6] US Food and Drug Administration. Initial decision. Withdrawal of approval of the new animal drug application for enrofloxacin for poultry [cited 2006 Mar 8]. Docket no. 00N-1571. p. 13–5. Available from http://www.fda.gov/ohrms/dockets/dailys/04/mar04/031604/00n-1571-idf0001-vol389.pdf
